# Reflective and Non-conscious Responses to Exercise Images

**DOI:** 10.3389/fpsyg.2017.02272

**Published:** 2018-01-11

**Authors:** Kathryn Cope, Corneel Vandelanotte, Camille E. Short, David E. Conroy, Ryan E. Rhodes, Ben Jackson, James A. Dimmock, Amanda L. Rebar

**Affiliations:** ^1^Physical Activity Research Group, School of Health, Medical and Applied Sciences, Central Queensland University, Rockhampton, QLD, Australia; ^2^Freemasons Foundation Centre for Men's Health, School of Medicine, The University of Adelaide, Adelaide, SA, Australia; ^3^Kinesiology and Human Development and Family Studies, The Pennsylvania State University, State College, PA, United States; ^4^Department of Preventive Medicine, Northwestern University, Chicago, IL, United States; ^5^Department of Kinesiology and Science, University of Victoria, Victoria, BC, Canada; ^6^School of Human Sciences, The University of Western Australia, Perth, WA, Australia

**Keywords:** automatic, reflective, perception, visual, image content, physical activity

## Abstract

Images portraying exercise are commonly used to promote exercise behavior and to measure automatic associations of exercise (e.g., via implicit association tests). The effectiveness of these promotion efforts and the validity of measurement techniques partially rely on the untested assumption that the images being used are perceived by the general public as portrayals of exercise that is pleasant and motivating. The aim of this study was to investigate how content of images impacted people's automatic and reflective evaluations of exercise images. Participants (*N* = 90) completed a response time categorization task (similar to the implicit association test) to capture how automatically people perceived each image as relevant to *Exercise* or *Not exercise*. Participants also self-reported their evaluations of the images using visual analog scales with the anchors: *Exercise*/*Not exercise, Does not motivate me to exercise*/*Motivates me to exercise, Pleasant*/*Unpleasant*, and *Energizing/Deactivating*. People tended to more strongly automatically associate images with exercise if the images were of an outdoor setting, presented sport (as opposed to active labor or gym-based) activities, and included young (as opposed to middle-aged) adults. People tended to reflectively find images of young adults more motivating and relevant to exercise than images of older adults. The content of exercise images is an often overlooked source of systematic variability that may impact measurement validity and intervention effectiveness.

## Introduction

Regular exercise is a major contributor to the maintenance of good health and wellbeing (Warburton et al., 2006; Stanton and Reaburn, 2014; Rebar et al., 2015). One-in-three adults in developed countries are not sufficiently active (Alford, 2010; World Health Organization, 2014), and investigations of the psychology of exercise can aid in efforts to improve global health. The use of images to represent exercise is common in the study of exercise, for example as a stimulus for testing attentional bias for exercise-related images (Berry et al., 2011). These exercise images are often selected based on untested assumptions of how these images are perceived or evaluated. The aim of this study is to provide empirical evidence for how the content of exercise images (e.g., age of people in the image, setting, activity) impacts people's reflective and non-conscious evaluations of images of exercise.

Evidence suggests that there is merit for interventions that target two types of psychological precursors to exercise—reflective and non-conscious processes (Chaiken and Trope, 1999; Evans and Frankish, 2009; Rebar et al., 2016a,b; Conroy and Berry, 2017). *Reflective processes* are intentional, slow, and include factors such as behavioral intentions, outcome expectancies, and perceived social norms. *Non-conscious processes* are unintentional, spontaneous behavioral influences and include factors such as habits, automatic evaluations, and automatic self-schemas (Rebar et al., 2016a). Images of exercise are sometimes used to measure people's non-conscious responses to exercise in instruments such as the implicit association test, as well as in interventions targeting both non-conscious and reflective exercise regulation (e.g., Markland et al., 2015; Antoniewicz and Brand, 2016).

Reflective processes are typically assessed via self-report; however, non-conscious processes are sometimes assessed using implicit measures (e.g., the implicit association task, lexical decision tasks), which use people's initial responses to stimuli (such as words or images) as a reflection of their non-conscious biases (e.g., Berry et al., 2008, 2011; Catitri et al., 2009). Notably, the validity of measures of non-conscious regulation of exercise, such as automatic evaluations, is partially reliant on the untested assumption that the stimuli being used are truly relevant to the concept of “exercise.” Although recent evidence exists regarding the *words and phrases* that people feel are most relevant to “exercise” (Rebar et al., 2016b), no study has tested what impacts people's evaluations of whether *images* are or are not relevant to “exercise.” Images can be more memorable and effective in conveying beliefs than words (McQuarrie and Phillips, 2005) and more easy to recall than text (Rayner et al., 2001). Additionally, images may have potentially potent motivational effects given that mental imagery of motor actions map onto the same brain areas as actual performance of the behaviors (Kreiman et al., 2000). Taking advantage of these positive impacts that images have on information processing may be a simple way to enhance the effectiveness of exercise promotion efforts.

Some studies have used images to enhance automatic evaluations of health behaviors using evaluative conditioning (e.g., Hollands et al., 2011; Markland et al., 2015; Bui and Fazio, 2016). For example, Markland et al. (2015) utilized exercise images in an implicit association test to determine whether presenting people with images of pleasant exercise experiences impacted either their automatic evaluations or reflective attitudes toward exercise and found that the imagery enhanced automatic evaluations but not reflective attitudes. Additionally, exercise images have been used in interventions targeting reflective processes such as public health advertising campaigns to raise awareness about the health benefits of exercise (Centers for Disease Control and Prevention, 2011; VicHealth, 2017) and web-based interventions for enhancing motivation to exercise (Vandelanotte et al., 2007; Davies et al., 2012). Although the use of exercise images is prominent throughout exercise interventions, very little is known about how characteristics of images might impact people's evaluations of how motivating, pleasant, or activating they are.

The content of an image can influence how a person processes the visual information of the image (Marchewka et al., 2014). Previous research from other fields suggests that the content of an image can influence how a person processes the visual information, depending on their non-conscious biases and/or reflections. Non-conscious biases are the initial evaluative and emotional reactions to images, which are then processed by reflective systems and translated into values, judgments, and decisions (Petty and Cacioppo, 1986; Chaiken and Trope, 1999; Gawronski and Bodenhausen, 2006; Cunningham and Zelazo, 2007; Evans and Frankish, 2009; Rothman et al., 2009). Importantly, non-conscious biases are not more or less “true” than reflective processes—they are simply different types of evaluations with unique influences on behavior (Gawronski and Bodenhausen, 2006). Given that exercise behavior can be impacted by these two levels of information processing, it is essential to investigate both the non-conscious and reflective evaluations of exercise images.

One characteristic of images that may impact people's evaluations may be the setting, given that people tend to find images of outside settings more vitalizing than images of inside settings (Ryan et al., 2010), and that exercising outside is more mood-enhancing than indoor exercising (Plante et al., 2007; Focht, 2009). The activity being performed in the exercise image may also impact people's evaluations. National and global health recommendations promote a range of exercise modalities including sport and recreation activities such as jogging, dancing, and swimming, as well as household tasks such as cleaning or gardening (The Department of Health, 2014; World Health Organization, 2017). To date, however, little is known about whether images of certain types of exercise activities are seen more favorably or motivating than others.

Another image characteristic that may influence evaluations of exercise images is whether the image includes an individual or a group. The so-called “cheerleader effect” suggests that images of people in groups are rated as more attractive than images of individuals (Walker and Vul, 2014) and may have underpinned previous findings showing that people respond more positively to images of people exercising in a group compared to alone (Burke et al., 2006). Evidence also suggests that people have more positive biases toward images of younger as opposed to older adults (Levy and Banaji, 2002), so it may be that the ages of people in exercise images impact people's evaluations of them. Additionally, the gender of people in the image may also impact people's non-conscious or reflective evaluations. Research suggests that there continues to be a societal bias in which portrayals of men are perceived as more relevant to the concept of exercise than women (Sherry et al., 2016). This society-level bias may permeate individuals' non-conscious reactions to exercise images, leading to general tendencies for people to automatically associate images of men with the concept of exercise more so than images of women.

The present study is the first to investigate how the content of exercise images impact people's non-conscious and reflective evaluations of the images. We hypothesized that people would have systematically different evaluations of exercise images as a function of the setting of the image, the activities being performed, whether the image was of a group or individual, and the age and gender of people in the image.

## Methods

### Participants and procedures

Participants (*N* = 90) were recruited from three public event locations in rural Queensland, Australia. They completed a survey on computers on site. Prior to completing the surveys, all participants provided informed consent and confirmed that they were 18 years or older. Following their involvement in the study, participants had the opportunity to enter into a random draw for one of four $200 AUD shopping vouchers.

The participants completed questions about demographic characteristics, current experience with walking, running, and vigorous activity (*not doing regularly/doing sometimes [less than once a week]/doing at least once a week*), and a response timed categorization task (to assess automatic associations of the images), and a self-report survey (to assess reflective evaluations of the images). The response categorization task and the self-report survey were presented in a counter-balanced order between participants in a 1:1 ratio. Overall, the task took between 20 and 40 min.

### Measures

#### Images

Each participant was presented with 98 randomly selected exercise images from a pool of 329. Forty-eight images were used to test automatic associations of exercise and another 50 were used to test reflective evaluations. No participant was presented with the same image twice. Images were sourced from Shutterstock and NAPS (Marchewka et al., 2014). The images were selected to include a variety of settings, group numbers, types of activity, and age and gender of the people in the image. The types of exercise activities portrayed in the images were selected based on the activities identified as most relevant to exercise to the general Australian population (e.g., walking, running, swimming, gardening, biking, sports; Rebar et al., 2016b). Further details of the images are available from the corresponding author.

#### Automatic associations with exercise

A response timed categorization task based on the Brief Implicit Association Test (BIAT; Sriram and Greenwald, 2009) involved the participants pressing either *E* or *I* on the keyboard to categorize the set of 48 exercise images and 48 control images of other behaviors (e.g., reading, standing outside, working at a computer) as “exercise” or “not exercise” as quickly and accurately as possible. Unlike the original BIAT, there were no attributes (pleasant/unpleasant) included in the categories. This is because the aim was to capture whether people automatically associated the image as exercise or not exercise, as opposed to capturing people's evaluative associations. Throughout the task, the button (*I* or *E*) that represented the categories switched four times to reduce the risk of learning effects. As is common for this test, the first two trials following each category switch were much longer durations compared to the other trials (i.e., learning effects) and so were omitted from analyses. The images (350 × 235 pixels) appeared one by one in the center of the screen until participants pressed one of the two buttons (*I* or *E*). If the wrong category was selected, a small red “X” appeared and the respondent had to correct their response by pressing the correct button. The response time to the correct response was recorded for each trial. The mean response time per image was used as an indication of how strongly people associated that image with the concept of exercise, with quicker times representing stronger automatic associations. In line with other response latency scoring procedures (e.g., Chevance et al., 2017) the top 10% of response times were truncated (*n* = 886, > 2,450 ms) prior to the calculation of the mean response time per image.

#### Reflective evaluations

Participants reported their reflective evaluations of the images using horizontal visual analog scales ranging from 0 to 100. Values were not visible to participants. The image was shown above the set of 4 items and remained on-screen while the participant used the mouse to move the arrow to the desired location on the scale. The arrow was initially set at the midpoint of the scale. Participants rated each of the 50 images on the scales with the anchors: *Exercise* to *Not exercise, Does not motivate me to exercise* to *Motivates me to exercise, Pleasant* to *Unpleasant*, and *Energizing/Activating* to *Calming/Deactivating*. Means were calculated for each of the four reflective evaluations per image.

### Image coding

Each image was coded independently by two of the authors based on a pre-determined list of image categories within five classification groups: *gender* (all men/all women/both), *age* (youth/young adult/adult/senior/mixed), *setting* (outside/inside), *group* (individual/couple/group), and *activity* (running/walking/swimming/surfing/bike riding/team sports/rowing/gym and exercise class/gardening/do-it-yourself work/housework/animal care and farm work). Interrater reliability was calculated as percentage agreement with a zero tolerance and unweighted Cohen's Kappa (κ; Gwet, 2014), with ≥ 0.80 representing strong agreement, 0.60 < κ < 0.79 representing moderate agreement, and 0.40 < κ < 0.59 representing weak agreement (McHugh, 2012). Then, discrepancies between coding were discussed and the code was agreed upon via collaboration of the two coders.

### Data analyses

A series of Analysis of Variance (ANOVA) tests and Tukey *post hoc* tests (if applicable) with Bonferroni corrections for multiple comparisons were conducted using *R* (Team R, 2013) to test whether there were systematic differences in people's non-conscious or reflective evaluations of the images based on image characteristics (i.e., gender, age, setting, group, and activity). With exception to the post-hoc test comparisons (adjusted for multiple corrections), alpha was set at *p* < 0.05. Effect sizes were calculated using Cohen's *d* (Cohen, 1992). Prior to analyses, some image characteristic categories had low cell sizes so were collapsed into other, relevant categories. Specifically, the age classification was collapsed into three groups—young adult/adult/senior, and the activities classification was collapsed into three groups: sport (running, walking, swimming, surfing, bike riding, team sports, rowing); gym (gym and exercise class); and active labor (gardening, DIY, housework, animal care/farm work).

## Results

### Intercoder reliability

Intercoder reliability between the two independent coders was strong for all characteristics across the images except *age*, which was moderate (κ = 0.59; agreement = 68.4). Reliability was highest for coding *group* (κ = 0.91; agreement = 96%) and *setting* (κ = 0.875; agreement = 94.5).

### Sample characteristics

The participants were mainly Australian adults (*n* = 88, 97.8%) with the majority from Queensland (*n* = 83, 92.2%). There was an equal sampling of gender (45 women, 50.0%), and a wide age range from 18–75 years (*M* = 38.17 years, *SD* = 16.5). Approximately a third of the sample had education at the level of a high school diploma (*n* = 31, 34.4%), or a bachelor or associate's degree (*n* = 29, 32.2%). Some had some university schooling but no degree (*n* = 15, 16.7%), and a few participants had education at less than high school level (*n* = 3, 3.3%). The majority of participants reported currently being employed or self-employed *(n* = 61, 67.8%). Some participants were students (*n* = 12, 13.3%), and 10% of participants were retired or pensioners (*n* = 9). Just over one third of participants (34.4%) reported an annual household income between AU$50,000 and AU$99,000 (*n* = 31), 22% (*n* = 20) reported an income between AU$100,000 and AU$149,000, while 17.8% *(n* = 16) reported income levels below AU$25,000. More than half the participants were married or in a domestic partnership (*n* = 51, 56.7%). About a third of the sample reported not regularly walking for ten consecutive min (*n* = 30, 31%), and slightly more reported walking regularly, at least once a week (*n* = 37, 41%). More than half reported not regularly running (*n* = 54, 60%), with only 9% of participants reporting regularly running (*n* = 8). Only 20% of participants reported engaging in no vigorous exercise (*n* = 18, 20%), and some reported engaging in 3 or more hours of vigorous exercise per week (*n* = 14, 14%).

### Image automatic associations with exercise

**Figure 1:**
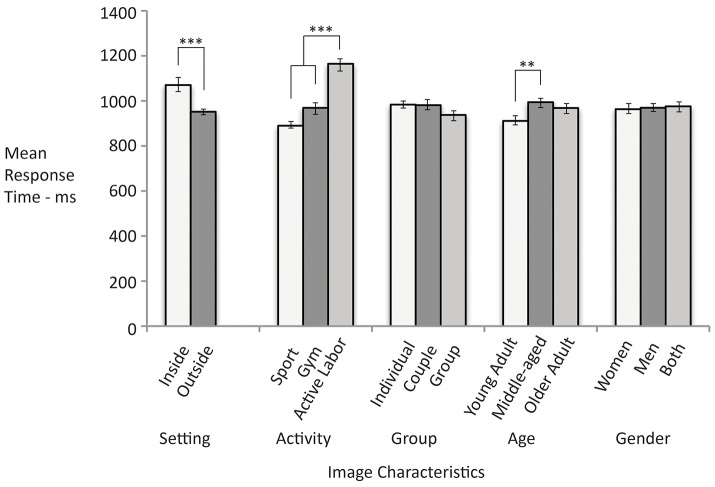
Faster response time indicates stronger automatic associations of the images with exercise. Significant differences are noted with asterisks. Error bars reflect standard errors.

Figure 1 presents the mean response times as a function of image characteristics including setting of the image, activities being portrayed, number of people in the image, and age and gender of people in the images. There were no significant differences in how quickly people responded to exercise images with groups, couples, or individuals, *F*_(2, 325)_ = 1.96, *p* = 0.14, or to images with women, men, or both, *F*_(2, 301)_ = 0.74*, p* = 0.90. However, people did respond faster to images as a function of setting, *F*_(1, 327)_ = 21.3, *p* < 0.001, activity, *F*_(2, 231)_ = 65.66, *p* < 0.001, and age of people in the image, *F*_(2, 220)_ = 5.47, *p* < 0.001.

People tended to have stronger automatic associations between the images and the concept of exercise if the images displayed an outside as opposed to an inside setting (*d* = 0.61). Also, people tended to have stronger automatic associations between the images and the concept of exercise if the images showed sport as opposed to gym-based (*d* = 0.60) or active labor (*d* = 1.73). People tended to more strongly automatically associate images with exercise if the images were of younger as opposed to middle-aged adults (*d* = 0.48). There was not a significant difference between the response times to images of young and older adults.

### Reflective evaluations of images

**Figure 2:**
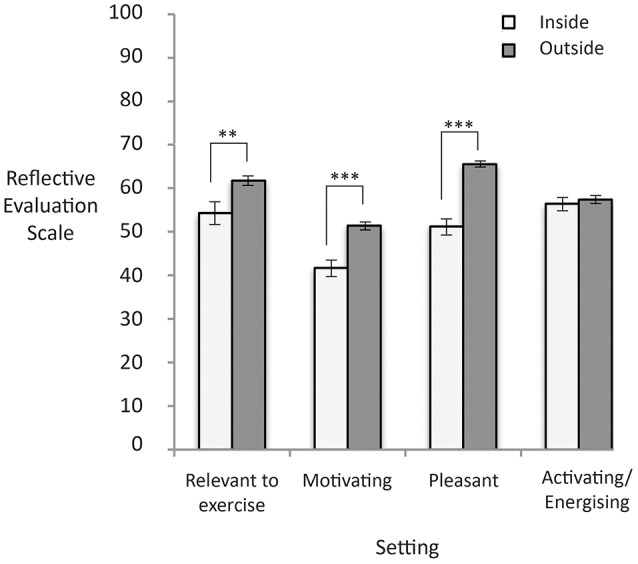
Higher scores represent higher reflective evaluation ratings on the 0–100 response scale. Significant differences are noted with asterisks. Error bars reflect standard errors.

Figure 2 shows the reflective evaluations based on image setting. There were significant differences between people's reflective response to the images as a function of the image characteristic of setting, *F*_(1, 327)_ = 9.38, *p* < 0.001, with people reporting exercise images set outside as being more relevant to exercise than images set inside, *d* = 0.43. Additionally, compared to images set inside, images set outside were reported to be significantly more motivating, *F*_(1, 318)_ = 23.82, *p* < 0.001, *d* = 0.72, and more pleasant, *F*_(1, 318)_ = 85.08, *p* < 0.001, *d* = 1.27. There were no significant differences in terms of how activating/energizing images were as a function of setting, *F*_(1, 318)_ = 0.25, *p* = 0.62.

**Figure 3:**
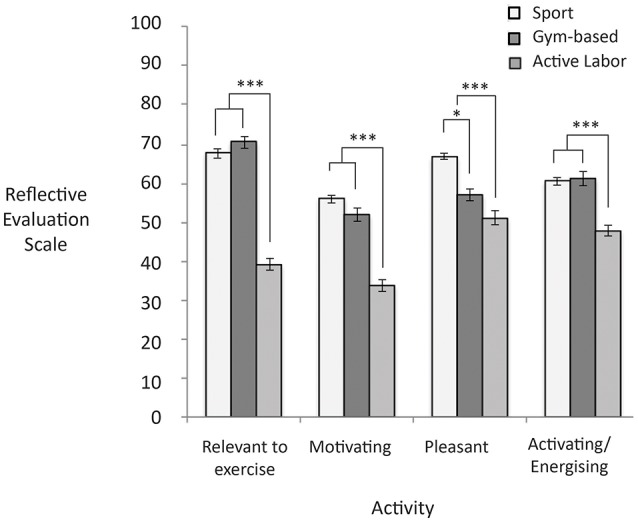
Higher scores represent higher reflective evaluation ratings on the 0–100 response scale. Significant differences are noted with asterisks. Error bars reflect standard errors.

Figure 3 shows the reflective evaluations as a function of activity type presented in the image. People tended to report that images of active labor activities were less relevant to exercise than images of gym-based (*d* = 3.23) or sport activities, *F*_(2, 231)_ = 115.9, *p* < 0.001, *d* = 2.42. Additionally, people tended to report that images of active labor activities were less motivating than images of gym-based (*d* = 1.80) or sport activities, *F*_(2, 231)_ = 79.74, *d* = 2.05; *p* < 0.001. People also tended to report that sport activity images were more pleasant than gym-based activity images (*d* = 1.08) and that active labor activity images were less pleasant than both gym-based (*d* = 0.54) and sport activity images, *F*_(2, 231)_ = 49.73, *p* < 0.001, *d* = 1.40. Additionally, people tended to report that images of active labor activities were less activating/energizing than images of gym-based (*d* = 1.38) and sport activities, *F*_(2, 229)_ = 26.11, *p* < 0.001, *d* = 1.23.

**Figure 4:**
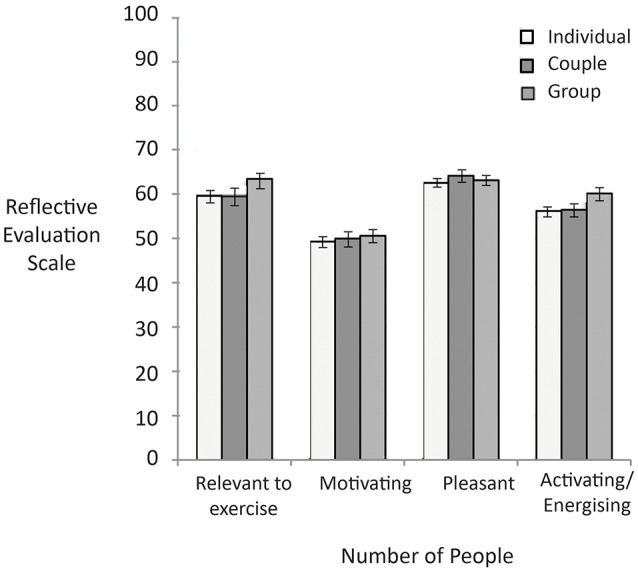
Higher scores represent higher reflective evaluation ratings on the 0–100 response scale. There were no significant differences. Error bars reflect standard errors.

Figure 4 shows the reflective evaluation scores based on number of people in the image. There were no significant differences in reflective evaluations of images as a function of whether the image included an individual, a couple, or a group (all *p'*s > 0.05).

**Figure 5:**
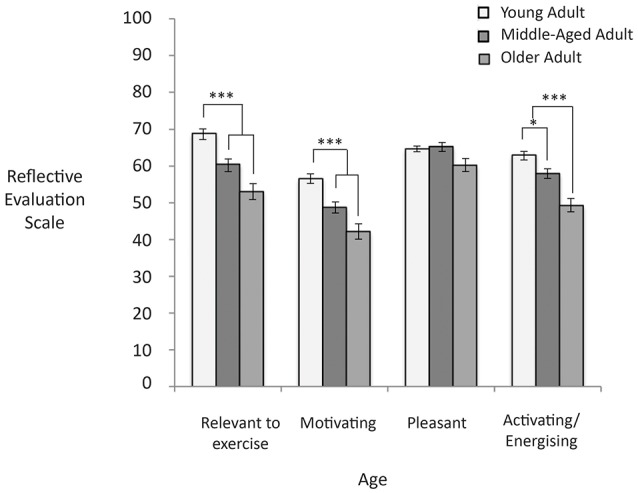
Higher scores represent higher reflective evaluation ratings on the 0–100 response scale. Significant differences are noted with asterisks. Error bars reflect standard errors.

Figure 5 shows the reflective evaluation scores based on age of people in the image. People tended to report images of young adults as more relevant to exercise than images of middle-aged (*d* = 0.48) and older adults, *F*_(2, 220)_ = 15.26, *p* < 0.001, *d* = 1.17. Additionally, people tended to report images of young adults as more motivating than images of middle-aged (*d* = 0.59) and older adults, *F*_(2, 220)_ = 12.75, *p* = 0.001, *d* = 0.87. People tended to report that images of older adults were less activating than images or middle-aged (*d* = 0.70) and young adults, *F*_(2, 220)_ = 16.86, *p* < 0.001, *d* = 1.24. There were no significant differences in how pleasant images were as a function of age, *F*_(2, 220)_ = 2.08, *p* = 0.13.

**Figure 6:**
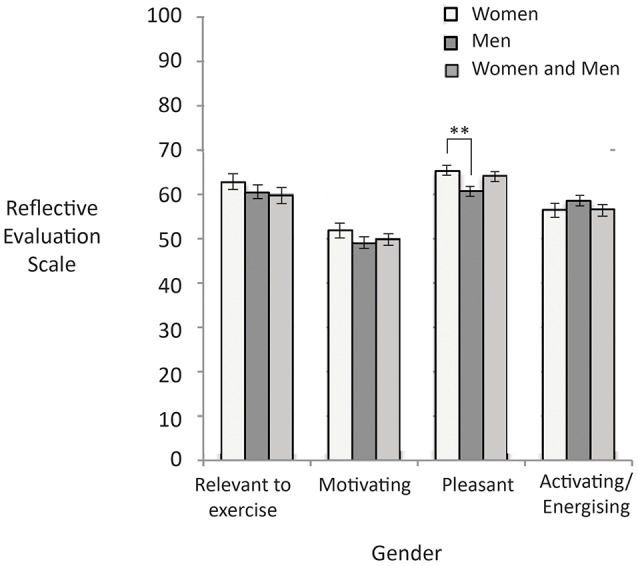
Higher scores represent higher reflective evaluation ratings on the 0–100 response scale. Significant differences are noted with asterisks. Error bars reflect standard errors.

Figure 6 shows the reflective evaluation scores as a function of the gender of people in the image. People tended to report images of women as more pleasant than images of men, *F*_(2, 301)_ = 4.99, *p* < 0.001, *d* = 0.41. However, there was no significant difference in how people rated images of women, men, or both in terms of how relevant images were to exercise *F*_(2, 301)_ = 0.74, *p* = 0.48, how motivating the images were *F*_(2, 301)_ = 1.19, *p* = 0.32, or how activating the images were *F*_(2, 301)_ = 0.89, *p* = 0.41.

## Discussion

The present study provides a comprehensive understanding of how image characteristics impact people's non-conscious and reflective evaluations of exercise images. In support of our hypotheses, the findings revealed that people had stronger automatic associations of images with exercise if the images displayed outside (as opposed to inside) settings, involved people playing sport or performing gym-based activities (as opposed to active labor), and were of young adults (as opposed to middle-aged or older adults). These findings were paralleled for the reflective evaluations—people reported that images were more relevant to the concept of exercise and motivated them to exercise more if they were set outdoors, of sport or gym-based activities, and included younger adults. Unexpectedly, images that were of groups versus individuals or men versus women had no impact on either non-conscious or reflective evaluations of how relevant people found images to be of exercise. The findings of this study also showed that exercise images were perceived as more pleasant if they were set outside and were of women. Images were rated as more activating if they were of sport or gym-based activities and young or middle-aged adults.

The setting of the exercise images had a significant impact on people's non-conscious and reflective evaluations of the images. These findings align with past evidence showing that outside exercise may have more substantial mood-enhancing effects than inside exercise (Plante et al., 2007) and the general preference toward images of natural environments (Ulrich, 1986; Franěk and Režný, 2017). When considering images for use in exercise interventions, it may be worthwhile to use outside settings when possible. Additionally, the setting of exercise images needs to be considered when in the measurement of non-conscious exercise regulatory processes. People will likely respond faster to exercise images set outside; however, this systematic bias may not be a bad thing. Including a variety of inside and outside exercise images may allow for a more comprehensive representation of the targeted concept of “exercise” (Nosek et al., 2006). Of note, the outside settings of the images in this study tended to include nature, as opposed to urban settings, so further research is needed to tease apart how people's evaluations differ across more nuanced setting types.

People tended to have less favorable evaluations of active labor activities compared to sport or gym-based activities. Encouraging active household chores or occupational work has its place in interventions as a way to aide people in overcoming barriers of perceived limited time for exercise; however, the findings of this study suggest it may be more encouraging for people to view images of sport or gym-based exercises. The present findings support previous evidence demonstrating that many people do not perceive active labor as relevant to exercise (e.g., Rebar et al., 2016b); therefore, consideration is needed for whether it is relevant to use images of active labor to assess or manipulate non-conscious regulation of exercise. Many of the active labor activity images used in this study depicted lower intensity of activity than the sport or gym based ones; therefore future studies should consider teasing apart people's evaluations of intensity as opposed to mode of activity in exercise images.

Whether images contained groups or individuals had no impact on people's non-conscious or reflective evaluations. Previous evidence suggests that group exercises are typically found to be more enjoyable than individual pursuits (Carnes and Barkley, 2015) and that people tend to have favorable biases toward images of groups as opposed to individuals (Burke et al., 2006; Walker and Vul, 2014). Preferences for group as opposed to individual programs can vary greatly between-people (Renjilian et al., 2001) and depend on characteristics of the group (e.g., Beauchamp et al., 2007), so it may be that preferences for individual or group exercise may be too idiosyncratic or personality-based to show consistent effects across studies (e.g., Courneya and Hellsten, 1998). Evidence suggests it may be most beneficial to encourage and measure exercise in both individual and social contexts (Burke et al., 2005).

Peopled tended to have stronger automatic associations of images to the concept of “exercise” if they were of young adults. An age bias was also prevalent in the reflective comparisons of this study, in that people tended to have biases toward exercise images of younger, as opposed to older, adults. These findings are in line with evidence of a general positivity bias toward youth (Levy and Banaji, 2002). There was also some support that people preferred images of women exercising as opposed to men, which seemingly opposes societal reflections of exercise as being a masculine type of behavior (Sherry et al., 2016). Although sub-group analyses were not possible within the present study, it is likely that these effects differ as a function of the individual's age and gender. People are more likely to relate to and prefer being presented with exercise intervention materials of people similar to themselves (Short et al., 2015; Jackson et al., 2017). Most likely, exercise images will be most effective if matched to the individual's own gender and age group. Overall, this study demonstrated that exercise images are not all perceived equally, and that there are systematic differences in how people perceive images depending on many image content characteristics.

This study provides new evidence about people's non-conscious and reflective evaluations of exercise images; however, future research is needed to extend on these findings to overcome some of the limitations. Specifically, the study had a relatively small, homogenous sample size so these findings should be tested in larger, more heterogeneous samples with sufficiently powered sub-groups to determine whether there are individual-level differences in perceptions of exercise images. Additionally, the study did not test how the use of different images impacted the effectiveness of behavior change, so experimental work is needed to investigate if these characteristics of images have differential impacts on exercise behavior. Previous research has demonstrated that images can be used to enhance health behaviors (e.g., Vandelanotte et al., 2007; Centers for Disease Control and Prevention, 2011; Hollands et al., 2011; Davies et al., 2012; Markland et al., 2015; Bui and Fazio, 2016; VicHealth, 2017). Further, some research suggests that images can activate implicit motives (Shantz and Latham, 2009; Rawolle et al., 2017), so future research should build on the potential for specific exercise images to enhance implicit motivation in interventions.

The present study provides empirical evidence for which types of exercise images may be most relevant to exercise and encouraging in these efforts. Globally, the findings suggest that images most relevant to the concept of “exercise” were those that were set outdoors, of sport or gym-based activities, and included younger adults. For large population-based promotion efforts, these may be the most effective types of images to represent exercise. For health practitioners or in delivery of interventions, tailoring images to be particularly relevant for the targeted population may be more worthwhile (Noar et al., 2007). For example, it may be activity websites, apps or wearable monitors could be tailored to use images based on user characteristics or preferences. Additionally, the systematic differences between which types of images people found most motivating, pleasant, and activating may be of use for trying to elicit certain exercise-relevant cognitive or affective states. For example, priming people with images perceived as pleasant and activating (i.e., of outdoor gym- or sport-based activities of young adults) may enhance people's automatic evaluations of exercise, which may lead to more exercise behavior (Conroy and Berry, 2017).

## Ethics statements

This study was carried out in accordance with the recommendations of Australia & National Statement on Ethical Conduct in Human Research, with written informed consent from all participants. All participants gave written informed consent in accordance with the Declaration of Helsinki. The protocol was approved by the Central Queensland University and Human Research Ethics Committee.

## Author contributions

KC and AR: Helped conceive of the idea of the study design, rated the images, analyzed the data, interpreted the findings, and provided intellectual content for the manuscript; CV, CS, DC, RR, BJ, and JD: Helped conceive of the idea of the study design, assisted in interpreting the findings, and provided intellectual content for the manuscript.

### Conflict of interest statement

The authors declare that the research was conducted in the absence of any commercial or financial relationships that could be construed as a potential conflict of interest.
